# The value of posterior colpotomy first technique on the vaginal length during total abdominal hysterectomy.

**DOI:** 10.1186/s12905-026-04476-1

**Published:** 2026-05-01

**Authors:** Mohamed N Farid, Bahaa M Hammad, Hany Saad, Asmaa AM Abdelfattah, Ahmed Mohamed Maged, Mohamed RM Soliman

**Affiliations:** 1 Department of Obstetrics and Gynecology, Kasr Al-Ainy Hospital, Cairo University, Cairo, Egypt; 2 Department of Obstetrics and Gynecology, Cairo university students’ hospital, Cairo, Egypt; 3 11 Eid Mostafa Street, Haram, 12111 Giza, Egypt

**Keywords:** Posterior colpotomy, Hysterectomy, Vaginal length, Sexual function

## Abstract

**Objective:**

To investigate the role of posterior colpotomy first technique in preserving vaginal length and support with total abdominal hysterectomy (TAH).

**Materials and methods:**

Eighty women candidate for TAH for benign lesion were randomly assigned to either classical technique (40 women) or posterior colpotomy first technique (40 women). The primary outcome parameter was the total vaginal length and shortening measured 3 months after the procedure.

**Results:**

The operative time was significantly longer in the posterior colpotomy group compared to classic group (106.28 ± 8.3 vs. 95.18 ± 18.35 min, *P* < 0.001). Total vaginal shortening was significantly lower in the posterior colpotomy group compared to classic group (0.99 ± 0.3 vs. 2.4 ± 0.8, *P* < 0.001). The number of women with postoperative dyspareunia was significantly lower in the posterior colpotomy group compared to classic group (4/40 (10%) vs. 8/40 (20%), *P* = 0.011). The female sexual function index score was significantly higher in the posterior colpotomy group compared to classic group (28.5 ± 2.5 vs. 24.19 ± 1.76, *P* = 0.038).

**Conclusion:**

The posterior colpotomy first technique is associated with less shortening of the total vaginal length, less occurrence of dyspareunia, and higher female sexual function index score compared to the classic approach for TAH.

**Supplementary Information:**

The online version contains supplementary material available at 10.1186/s12905-026-04476-1.

## Introduction

The most common major surgical operation in gynecology is hysterectomy. Over 40% of women are predicted to get a hysterectomy by the time they are 64 years old. According to Wilcox et al. [1], 13–37% of women who have a hysterectomy say that their sexual function has declined.

However, according to some findings, a hysterectomy could enhance a woman’s sexual function. According to some research, a hysterectomy-induced reduction in vaginal length may result in dyspareunia and negatively impact female libido. The vagina is cylindrical, and there are many local nerve endings at the vaginal apex. Therefore, the adverse impact on sexual function may be more severe due to the extra tissue lost from the vaginal apex during the creation of the vaginal cuff following hysterectomy [2].

Since the cervix (3–4 cm in diameter) enters the vagina via the anterior vaginal wall, the posterior vaginal wall is longer than the anterior vaginal wall. Women may vary in their vaginal length (VL), breadth, and size. The anterior vaginal wall ranges widely in length from 4.4 to 8.4 cm, with an average of 6.3 cm. The posterior vaginal wall has an average length of 9.8 cm, ranging from 5.1 to 14.4 cm. Height, weight, menopause, hysterectomy, and reconstructive surgery are some of the variables that might impact vaginal length [3].

The diameter of the cervix may range from 3 to 4 cm. A much shorter vagina is thought to result from the removal of this structure and subsequent stitching of the anterior and posterior vaginal walls together. The distance between the introitus and the posterior fornix is often equal to the entire vaginal length when a cervix is present [4].

The vaginal length measured to the pouch of Douglas appears to remain relatively undisturbed along the posterior vaginal wall, which is likely why removing a cervix with a diameter of 3–4 cm from the anterior vaginal wall does not appear to result in a corresponding 3–4 cm decrease in vaginal length [3].

The primary hysterectomy procedure that may regulate vaginal length is colpotomy. Following bladder mobilization, uterine artery ligation, and uterosacral ligament cutting, the traditional method for completing abdominal hysterectomy is anterior vault colpotomy. The posterior colpotomy procedure involves cutting the vaginal wall from the topmost portion of the uterosacral ligaments, then circumferentially completing the colpotomy at the uppermost portion of the vagina while preserving the paracervical ligaments as much as possible. This method prevents vaginal lengthening and maintains the vagina’s apical support [5].

This study aimed to investigate the role of (posterior colpotomy first) technique in preserving vaginal length and support with total abdominal hysterectomy.

## Materials and methods

This double blind randomized controlled study was conducted at Kasr Al-Ainy maternity hospital, Cairo university in accordance with the Declaration of Helsinki ethical standards and following the CONSORT guidelines between March 2023 and March 2024. The study was approved by kasr Alainy ethical committee (MD-118-2023). The trial was registered retrospectively at ClinicalTrials.gov, with NCT07308197 number.

Eighty women candidates for total abdominal hysterectomy for benign lesions were recruited. The research included women between the ages of 40 and 65 years who were sexually active, had a BMI of 25–40 kg/m2, had no previous abdominal surgery, and had a benign reason for a hysterectomy (multiple fibroid uterus, adenomyosis, or endometrial hyperplasia). Patients who had vaginal and uterine prolapse, subtotal hysterectomy, caesarean hysterectomy in patients with major obstetric hemorrhage (placenta previa, placenta accreta spectrum, uncontrolled postpartum hemorrhage), or malignant indications of total abdominal hysterectomy (uterine and cervical carcinoma) were not included in the study.

The comparison of vaginal length between women having abdominal hysterectomy utilizing the posterior colpotomy 1st method and those receiving the traditional anterior colpotomy 1st approach was used to determine the sample size. According to a similar study that substituted a total laparoscopic hysterectomy for the posterior colpotomy first technique [2], the mean ± SD of vaginal length in the posterior colpotomy 1st group was 9.4 ± 1.3 cm, whereas it was 8.7 ± 1.1 cm in the classical anterior colpotomy 1st group. Therefore, using Student’s t test for independent samples, we determined that the minimum appropriate sample size was 40 women in each group to be able to reject the null hypothesis with 80% power at α = 0.05 level. PS Power and Sample Size Calculations Software, version 3.1.2 for MS Windows (William D. Dupont and Walton D., Vanderbilt University, Nashville, Tennessee, USA), was used to calculate the sample size.

Computer-generated allocation sequences were used for randomization. With random numbers printed in opaque envelopes, this sequence was organized in a table with two groups. By selecting one of the envelopes, the patient was assigned, and the number inside the envelope was chosen at random for her group. Group A (Classic technique): the traditional hysterectomy was performed on forty patients. Forty patients had a hysterectomy using the posterior colpotomy first approach (Group B). Following an explanation of the purpose, nature of the research, risks and benefits and their right to withdraw from the trial at any stage, all participants gave their informed written consent. The following information was gathered for each patient in the patient group. Both the participants and outcome assessor were blinded while the operator was not blinded because of the nature of the technique.

Every participant had a thorough medical history as well as pelvic and general exams. Six specific sites in the vagina are used to assess any vaginal or uterine prolapse on the POP-Q system: points Aa and Ba for the anterior vagina, Ap and Bp for the posterior vagina, and C and D for the cervix/vault. To elicit the POP as much as possible, the patient is urged to stretch, preferably while standing. After that, the designated spots’ locations are measured in relation to the hymenal ring and noted on a grid [6].

Imaging and laboratory evaluation (transvaginal ultrasound). Using a closed ring forceps under the surgeon’s fingers, the vaginal length was measured from the hymenal ring to the posterior vaginal fornix. Centimeters were used to measure the distance and record the location where the instrument crossed the hymenal ring.

All the procedures were conducted using the same surgical technique with a team of surgeons having close surgical experience. A lower midline or Pfannenstiel incision is the first step in the complete abdominal hysterectomy procedure. They clamp, cut, and ligate the top pedicles. The cardinal and uterosacral ligaments were dissected, the wide ligament’s leaves were cut, the bladder was carefully lowered, and the uterine vessels were skeletonized, cut, and tied. In group A (traditional technique): the surgeon makes a circumferential incision after initially entering the anterior vaginal wall while in Group B (posterior colpotomy first) approach, entering the vagina via the posterior wall at the uppermost part of the uterosacral ligaments was done, followed by the left lateral fornix then completed the incision anteriorly.

Reconstruction of the pelvic floor to ensure hemostasis and good pelvic support. The operative time was assessed. The primary outcome parameter was total vaginal length and shortening measured at 3 months postoperative. As point D on the POP-Q system is omitted after hysterectomy, point C level was reassessed.

Total vaginal shortening (TVS) and vaginal shortening ratio (VSR) were calculated according to the formulas given below.

$$\mathrm{TVS}=\mathrm{Preoperative}\;\mathrm{VL}-\mathrm{Postoperative}\;\mathrm{at}\;\text{ 3 months}$$

$$\mathrm{VSR}=\mathrm{Preoperative}\;\mathrm{VL}-\mathrm{Postoperative}\;\mathrm{at}\;\text{ 3 months}\;/\mathrm{Preoperative}\;\mathrm{VL}\times100$$

Secondary outcomes included operative time, newly developed dyspareunia and assessment of postoperative sexual function using female sexual function index. The presence of newly formed dyspareunia was evaluated in relation to postoperative vaginal length. A questionnaire measuring desire, arousal, lubrication, orgasm, pleasure, and pain was used to measure the female sexual function index (FSFI) three months after surgery [7]. Sexual desire, arousal, lubrication, orgasm, pleasure, and pain are the six domain scores in this 19-item scale that assesses sexual function over the previous four weeks [8].

The FSFI has excellent psychometric qualities. Additional psychometric research has been conducted on the FSFI since its creation in 2000, confirming the original component structure as well as its validity and reliability. Individuals with a total FSFI score of less than 26 will be deemed at risk for sexual dysfunction and will need further evaluation. The English version of the FSFI is widely used in therapeutic studies and is quickly becoming as the gold standard for assessing women with sexual issues [9]. Anis et al. [10] have confirmed the Arabic version.

### Statistical analysis

Microsoft Excel for Windows Office 2019 was used to gather, code, and insert the data into a spread sheet. Version 21.0 of the Statistical Package of Social Science (SPSS) software is used to analyze data. The data was shown in tables and graphs, and quantitative data such as mean and standard deviation were compared using the t-test. The qualitative data presented as numbers and percentages were compared using the chi-square test. When *p* < 0.05, the P value is regarded as significant.

## Results

**Fig. 1:**
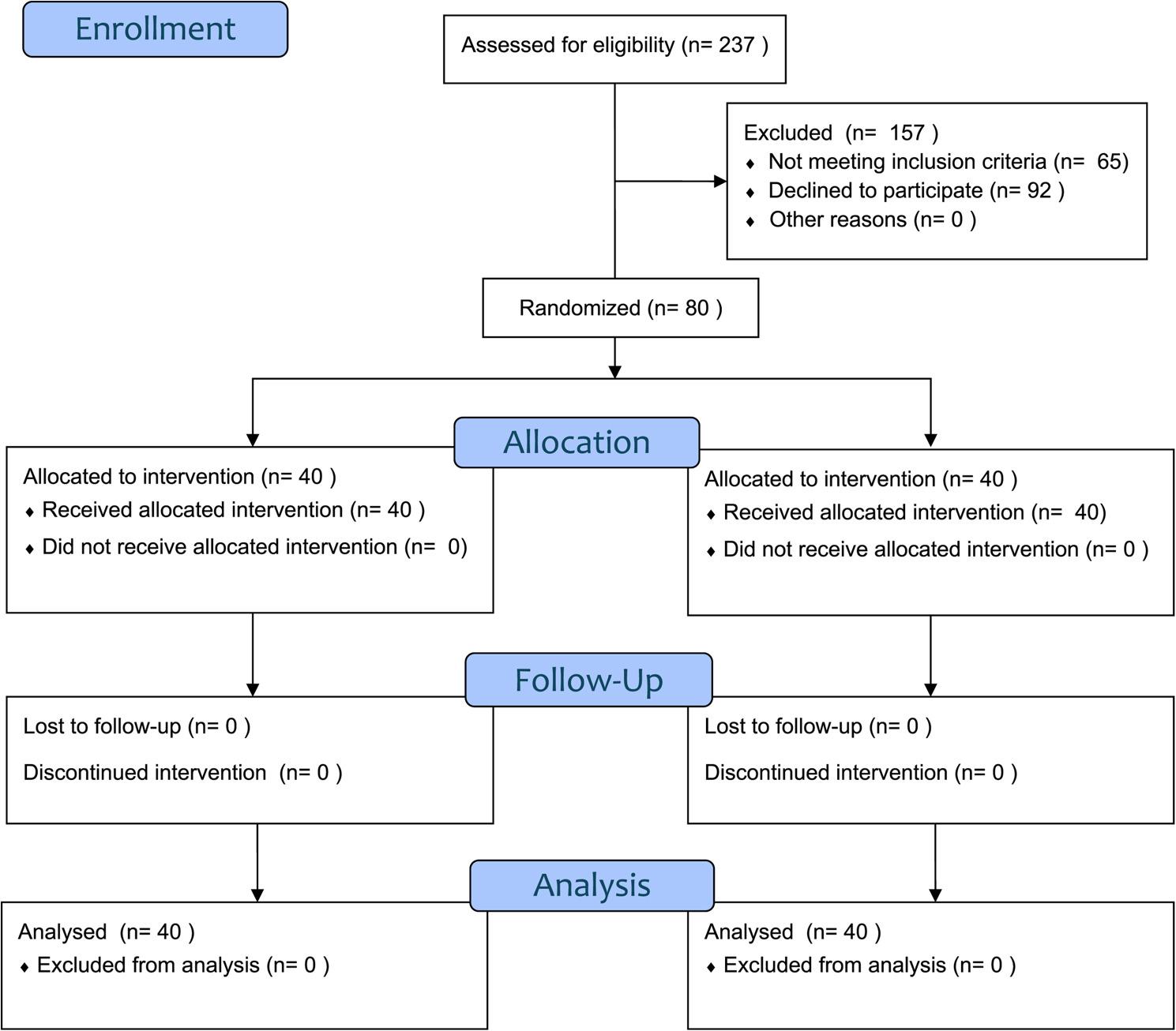
Consort flow diagram

Figure 1 showed the consort flow chart of the study.

Both groups were comparable as regard age, parity, body mass index, mode of delivery, indications for the operation and laboratory investigations (Table 1).

**Table 1:** Characteristics of the study population

		Classic hysterectomy (*n* = 40)	Posterior colpotomy first (*n* = 40)	*P* value
Age (years)		51.6 ± 8.0	51.8 ± 8.3	0.913
Parity		2.75 ± 1.0	2.7 ± 1.1	0.836
BMI (kg/m2)		31.1 ± 5.5	31 ± 5.9	0.904
Mode of delivery	VD	12 (30%)	9 (22.5%)	0.446
	CD	28 (70%)	31 (77.5%)	
Indication for hysterectomy	Fibroid uterus	19 (47.5%)	15 (37.5%)	0.765
	Adenomyosis	10 (25%)	15 (37.5%)	
	Endometrial hyperplasia	11 (27.5%)	10 (25%)	
Laboratory Investigations	Hemoglobin (gm/dL)	11.8 ± 0.93	11.9 ± 1.14	0.504
	WBCs count (X10^3^)	7.8 ± 1.7	7.9 ± 1.8	0.838
	Platelet count (X10^3^)	231.4 ± 19.7	233.9 ± 18.5	0.389
	PT (seconds)	11.8 ± 0.8	12 ± 0.8	0.979
	INR	1.2 ± 0.2	1.2 ± 0.2	0.522

Data are presented as mean ± SD or number (percentage)

*BMI* Body mass index, *CD* Cesarean delivery, *INR* International normalized ratio, *PT* Prothrombin time, *VD* Vaginal delivery, *WBCs* White blood cells

Women in the posterior colpotomy group had significantly longer operative time, longer total vaginal length, less total vaginal shortening, and less dyspareunia compared to women in the classic group (Table 2).

**Table 2:** outcome parameters

		Classic hysterectomy (*n* = 40)	Posterior colpotomy first (*n* = 40)	*P* value
Operative time (minutes)		95.18 ± 18.35	106.28 ± 8.3	0.001
Total vaginal length (cm)	Preoperative	10.58 ± 1.77	10.73 ± 1.6	0.692
	Postoperative	8.4 ± 1.2	9.7 ± 1.1	0.002
	*P* value	< 0.001	0.025	
TVS		2.4 ± 0.8	0.99 ± 0.3	< 0.001
VSR		16.1 ± 4.6	8.44 ± 3.8	< 0.001
Dyspareunia	Preoperative	1(2.5%)	2(5%)	0.905
	Postoperative	8(20%)	4(10%)	0.011
	*P* value	< 0.001	0.099	
FSFI	Desire	3.26 ± 0.96	3.8 ± 0.5	0.208
	Arousal	3.59 ± 0.57	4.1 ± 0.3	0.381
	Lubrication	2.97 ± 0.45	3.5 ± 0.7	0.021
	Orgasm	3.39 ± 0.89	4.8 ± 0.5	< 0.001
	Satisfaction	3.62 ± 0.81	3.8 ± 0.5	0.089
	Pain	2.37 ± 0.54	4 ± 0.6	< 0.001
	Total score	24.19 ± 1.76	28.5 ± 2.5	0.038
	FSD	10(25%)	5(12.5%)	0.024

Data are presented as mean ± SD or number (percentage)

*FSD* Female sexual dysfunction, *FSFI* Female sexual function index, *TVS* Total vaginal shortening, *VSR* Vaginal shortening rate

Regarding FSFI, women in classic hysterectomy group had lower mean of all domains than group B but with statistically significant difference in lubrication, orgasm, and pain and total FSFI score compared to posterior colpotomy group. Regarding total score, 25% in classic group had sexual dysfunction versus 12.5% in posterior colpotomy group (Table 2).

Women with dyspareunia had a significantly shorter total vaginal length when compared to other women (5.08 ± 0.9 vs. 9.85 ± 1.62 cm, respectively, *P* < 0.001).

Table 2 showed that Vaginal length was statistically significantly shorter in patients with dyspareunia (*p* < 0.001).

**Fig. 2:**
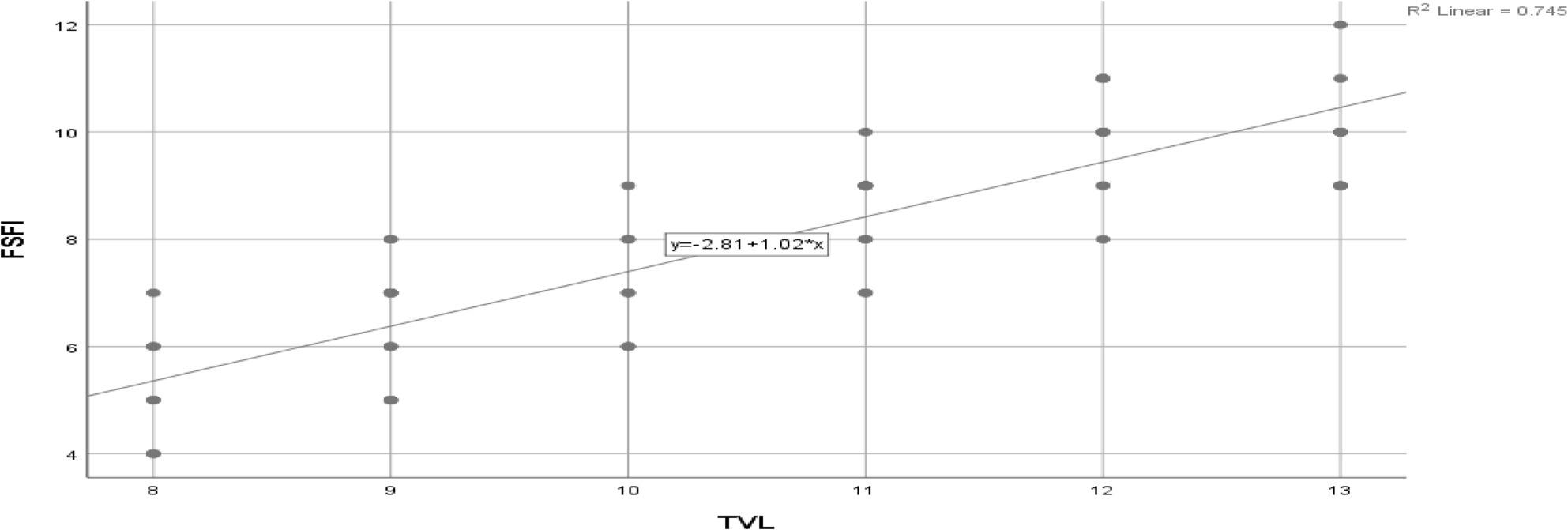
Correlation between TVL and FSFI

There was a statistically significant direct weak correlation between TVL with lubrication, orgasm, pain and total FSFI as in Table 3; Fig. 2.

**Table 3:** Correlation between total vaginal length and Female sexual function index

	Total vaginal length	
	*r*	*P* value
Desire	0.071	0.817
Arousal	0.108	0.341
Lubrication	0.212	0.033
Orgasm	0.187	0.044
Satisfaction	0.116	0.461
Pain	0.281	0.023
FSFI score	0.341	0.002

Pearson correlation

## Discussion

This study found that the posterior colpotomy first hysterectomy is associated with less shortening of the total vaginal length, less incidence of dyspareunia, better FSFI score especially in lubrication, orgasm and pain items when compared to the classic hysterectomy technique.

The impact of a hysterectomy and/or the kind of hysterectomy done on sexual life is so significant that it cannot be disregarded, especially because 85% of patients have an active sexual life. Loss of female genital organs, loss of nerve tissue, reduction in blood supply, loss of lubrication due to cervix loss, and adverse effects of scar tissue have all been linked to an increase in sexual dysfunction in women who have had hysterectomy [11].

According to some studies conducted in recent years, the elimination of organic issues that negatively impact sexual function following a hysterectomy has resulted in a notable improvement in both sexual function and quality of life [12]. Women’s vaginal length (VL), breadth, and size may vary from one another. According to certain theories, sexual function may be impacted by variables that alter vaginal length, such as height, weight, menopause, hysterectomy, or reconstructive surgery [13].

Accordingly, some research has shown that the reduced vaginal length after a hysterectomy may result in dyspareunia and negatively impact female libido. There is debate on the connection between postoperative vaginal length and the kinds of hysterectomy [2].

We believe that the more accurate identification of the colpotomy site and improved vision of the vaginal fornix via the use of the uterine manipulator are the causes of the lower TVS and VSR in the TLH group. The removal of extra tissue from the vagina as a result of identifying the colpotomy region using digitals might be the cause of the increased incidence of VSR and TVS in TAH.

In a standard total abdominal hysterectomy, colpotomy is ultimately completed circumferentially at the cervicovaginal junction. However, the sequence of incision (anterior vs. posterior) may still influence the *functional plane* of dissection and the final level of transection through indirect technical factors. Initiating colpotomy anteriorly versus posteriorly can alter the direction of traction, exposure, and the surgeon’s visual landmarks, particularly in the context of bladder reflection anteriorly and rectovaginal space development posteriorly. These differences may subtly shift the effective dissection plane, especially under variable tissue tension, potentially resulting in small but measurable differences in residual vaginal length. The statistically significant observed difference in vaginal length may or may not represent a clinically meaningful effect in isolation.

In a retrospective analysis, there was no discernible change in sexual function between the groups, despite the vaginal length being lower in VH than in robotic hysterectomy [14].

Patients who had greater VL preservation with a uterine manipulator had higher postoperative sexual function ratings, according to prospective research by Kiyak et al. [15]. According to a recent meta-analysis, VL decreased after a hysterectomy, although it was unrelated to sexual dysfunction or dyspareunia [16]. As is evident from the research, there is debate and uncertainty about the effects of different hysterectomy types on vaginal length and sexual function.

VL measurement is the foundation of current research in literature. Vaginal breadth, length, and size, however, might vary from woman to woman. Menopausal status, height, and weight are all known to have an impact on VL. The vagina is cylindrical, and there are many local nerve endings at the vaginal apex. Therefore, the adverse impact on sexual function may be more severe due to the extra tissue lost from the vaginal apex during the creation of the vaginal cuff after hysterectomy [15].

How sexual function will be impacted is one of the most crucial concerns that patients and physicians need to know the answers to following hysterectomy. According to reports, vaginal shortening may result in dyspareunia and impact sexual function in addition to factors that affect sexual function in the postoperative period, including age, socioeconomic status, presence of oophorectomy, marital status, preoperative sexual function, operation indication, and surgical technique [17].

Our findings are consistent with another research that found that 6% of patients had dyspareunia after a complete abdominal hysterectomy [18].

Patients who had complete hysterectomy saw more vaginal tissue loss than those who had partial hysterectomy. Together with the notable decline in FSFI scores seen in the total hysterectomy group relative to the subtotal hysterectomy group, this tissue loss may be linked to the loss of vaginal erogenous zones and nerve damage that have been previously proposed in the literature [19].

The need to clarify the connection between vaginal morphology and sexual function is heightened by contradictory findings in research examining sexual functioning after hysterectomy. Patients’ FSFI ratings significantly increased after a hysterectomy, according to Dedden et al. However, Flory et al. [20] were unable to find a significant difference in sexual function between subtotal hysterectomy and complete hysterectomy. Patients with conditions including endometriosis and persistent pelvic discomfort, which may significantly impair sexual function, were enrolled in these investigations. The patients’ sexual dysfunctions were assessed using their pre-operative FSFI scores. Despite the loss of vaginal tissue, hysterectomy may have had a favorable impact on sexual function by removing the detrimental consequences of these disorders. The lack of preoperative FSFI scores and the larger number of patients at menopause made it impossible to assess preoperative sexual life in this research. Furthermore, we did not assess the patients’ marriages or sexual relationship dysfunction, which may have led to misunderstandings.

This study is the first study to evaluate the effect of posterior colpotomy first technique on vaginal length and sexual function. Its main strengths are related to its design as randomized controlled one with double blinding of both participants and outcome assessor, adequately predetermined sample size, proper selection through strict inclusion and exclusion criteria.

This study is not without limitations. The main limitation was the absence of long term follow up to find the long term sequalae of the procedure. Other limitations included being a single center study and lack of operator blinding. Non-reporting of the preoperative FSFI could appear as a limitation, although none of the included women reported any preoperative sexual problems apart from 3 women who had preoperative dyspareunia (1 in classic hysterectomy and 2 in posterior colpotomy groups). The relatively small sample size may limit robustness of dyspareunia outcomes.

When a woman needs a complete hysterectomy for a benign condition, the posterior colpotomy first technique may be better than the traditional abdominal way, especially in terms of minor peri-operative problems, blood loss, and hospital stay. Longer operating times seem to be the sole compromise, although bigger research is desperately required to validate the study’s findings.

In conclusion we found that patients who had postoperative dyspareunia had shorter vaginal lengths than those who did not; the typical complete abdominal hysterectomy considerably shortens the vaginal length compared to the posterior colpotomy first procedure. Compared to the posterior colpotomy first approach, postoperative dyspareunia is more prevalent after a conventional hysterectomy.

Among properly qualified gynecologists, the posterior colpotomy first approach in abdominal hysterectomy has become a feasible substitute for the traditional complete abdominal hysterectomy.

## Supplementary Information

Supplementary Material 1.

## Data Availability

All data are available and can be supplied by all authors upon reasonable request.
